# Analyzing the impact of feature selection methods on machine learning algorithms for heart disease prediction

**DOI:** 10.1038/s41598-023-49962-w

**Published:** 2023-12-18

**Authors:** Zeinab Noroozi, Azam Orooji, Leila Erfannia

**Affiliations:** 1 Department of Artificial Intelligence, Islamic Azad University of Kazeroon, Kazeroon, Iran; 2 Department of Advanced Technologies, School of Medicine, North Khorasan University of Medical Sciences (NKUMS), Bojnurd, North Khorasan Iran; 3 Health Human Resources Research Center, Clinical Education Research Center, Shiraz University of Medical Sciences, Shiraz, Iran; 4 Health Information Management Department, School of Health Management and Information Sciences, Shiraz University of Medical Sciences, Shiraz, Iran

**Keywords:** Computational biology and bioinformatics, Data mining

## Abstract

The present study examines the role of feature selection methods in optimizing machine learning algorithms for predicting heart disease. The Cleveland Heart disease dataset with sixteen feature selection techniques in three categories of filter, wrapper, and evolutionary were used. Then seven algorithms Bayes net, Naïve Bayes (BN), multivariate linear model (MLM), Support Vector Machine (SVM), logit boost, j48, and Random Forest were applied to identify the best models for heart disease prediction. Precision, F-measure, Specificity, Accuracy, Sensitivity, ROC area, and PRC were measured to compare feature selection methods' effect on prediction algorithms. The results demonstrate that feature selection resulted in significant improvements in model performance in some methods (e.g., j48), whereas it led to a decrease in model performance in other models (e.g. MLP, RF). SVM-based filtering methods have a best-fit accuracy of 85.5. In fact, in a best-case scenario, filtering methods result in + 2.3 model accuracy. SVM-CFS/information gain/Symmetrical uncertainty methods have the highest improvement in this index. The filter feature selection methods with the highest number of features selected outperformed other methods in terms of models' ACC, Precision, and F-measures. However, wrapper-based and evolutionary algorithms improved models' performance from sensitivity and specificity points of view.

## Introduction

The prevalence of cardiovascular disease is on the rise worldwide such that the World Health Organization (WHO) estimates that 17 million people die annually from cardiovascular diseases, particularly stroke and heart attack. These diseases are responsible for 31% of global mortality and are considered the primary cause of death worldwide. It is estimated that the death rate from cardiovascular disease will rise to 22 million people by 2030. According to American Heart Association statistics, 50% of adults in the United States suffer from cardiovascular disease^1–3^. Risk factors include lifestyle behaviors, age, gender, smoking; family history, obesity, high blood fat, blood sugar level, poor food, alcohol consumption, and body weight are all factors that might contribute to these disorders, which are brought on by the heart's abnormal functioning. It is crucial to recognize the behaviors and warning symptoms of cardiovascular disorders^1^. Several tests, including auscultation, ECG, blood pressure, fat, and blood sugar, are required to diagnose CVD. Prioritizing these tests is crucial since they might often take a long time to complete while the patient has to start taking his/her medication right away. It's critical to recognize the numerous healthy behaviors that contribute to CVD^4^. On the other hand, this condition is challenging to identify because of the numerous risk factors that contribute to its onset. The survival rate of patients can be increased by timely and accurate diagnosis of certain disorders, though^2^.

A proper diagnosis is essential to the functioning of the health system. In the US, 5% of outpatients are given a serious medical illness that is misdiagnosed. This problem not only puts the patient in danger, but also leads to ineffective diagnostic procedures and other inefficiencies in the healthcare system. Diagnostic mistakes raise the expense of the healthcare system and erode public confidence in it. On the other hand, a lot of healthcare professionals are dissatisfied with the amount of time therapists spend entering data into computers, which reduces the effectiveness of doctor-patient contact^5^. The diagnosis of a heart attack is a highly complex and important procedure that must be conducted with care and precision. It is typically based on the knowledge and experience of the physician, which, if not done properly, can result in significant financial and life-altering expenses for the patient^6^. However, not all physicians possess the same expertise in subspecialties, and the geographical distribution of qualified specialists is uneven. As a result of these multiple factors used to evaluate the diagnosis of the heart attack, physicians typically make the diagnosis based on the patient's present test results^7^. Additionally, doctors review prior diagnoses made on other patients who have similar test results. These intricate procedures are, however, of little importance^8^.

To accurately diagnose heart attack patients, a physician must possess expertise and experience. Consequently, the obligation to leverage the knowledge and expertise of various professionals and the clinical screening data collected in databases to facilitate the analysis process is seen as a beneficial framework that integrates clinical selection aids and computer-aided patient records. Furthermore, it can reduce treatment errors, enhance patient safety, eliminate unnecessary conflicts, and enhance patient outcomes. Machine learning has been extensively discussed in the medical field, particularly for the diagnosis and treatment of diseases^7^. Recent research has highlighted the potential of machine learning to improve accuracy and diagnostic time. AI-based tools constructed with machine learning have become increasingly effective diagnostic tools in recent years^9,10^. Machine learning algorithms are highly effective in predicting the outcome of the data in a large amount. Data mining is a process of transforming large amounts of raw data into data that will be highly useful for decision-making and forecasting^11^. By producing more precise and timely diagnoses, machine learning technology has the potential to transform the healthcare system and provide access to quality healthcare to unprivileged communities worldwide. Machine learning has the potential to shorten the time it takes for patients to meet with their physicians, as well as to reduce the need for unnecessary diagnostic tests and enhance the precision of diagnoses. Preventive interventions can significantly reduce the rate of complex diseases^1,2^. As a result, many clinicians have proposed increasing the identification of patients through the use of Machine Learning and predictive models to reduce mortality and enhance clinical decision-making. Machine learning can be used to detect the risk of cardiovascular disease and provide clinicians with useful treatments and advice for their patients^12^.

In addition to the various cardiovascular disorders, there are pathological alterations that take place in the heart and the blood vessels. Data classification can enable the development of tailored models and interventions that reduce the risk of cardiovascular disease. These analyses assist medical professionals in re-evaluating the underlying risks and, even if a prior vascular disease has occurred, can provide more efficient solutions and treatments to improve the quality of life and extend life expectancy^13^, and reduce mortality. An expert can use supervised learning to answer the following: whether a medical image contains a malignant tumor or a benign tumor. Is a patient with heart disease likely to survive? Is there a risk of disease progression? Is it possible for a person with heart disease to develop heart disease with existing factors? These and other questions can be answered using supervised learning techniques and classification modeling^14,15^. Classification is one of the most common methods used in data mining. It divides data into classes and allows one to organize different kinds of data, from complex data to simple data. Classification is one of the supervised learning methods in data mining. The main goal of classification is to connect the input variables with the target variables and make predictions based on this relationship. The classification techniques used in this study ranged from decision tree to support vector machines (SVM) and random forest (Random Forest)^16^. In a study conducted by Melillo and colleagues, the CART algorithm was found to have the highest accuracy of 93.3% among the other algorithms. This algorithm was used to determine which patients had congestive heart disease, and which patients were at lower risk^17^.

Although Machine Learning (ML) is essential for the diagnosis of a wide range of diseases, the production of large-scale data sets and the presence of numerous non-essential and redundant features in these data sets is a significant deficiency in ML algorithms^8^. Furthermore, in many cases, only a small number of features are essential and pertinent to the objective. As the rest of the features are disregarded as trivial and redundant, the performance and accuracy of the classification are adversely affected. Therefore, it is essential to select a compact and appropriate subset of the major features to enhance the classification performance, as well as overcome the "curse of dimensionality". The purpose of feature selection techniques is to assess the significance of features. The aim is to reduce the number of inputs for the requirements that are most pertinent to the model. In addition to reducing the number of inputs, feature selection also significantly reduces the processing time. Even if several feature selection techniques have been employed in decision support systems in medical datasets; there are always improvements to be made^18^.

Previous research on predicting heart disease in two broad categories has focused on either optimizing algorithms based on various machine learning techniques or attempting to optimize algorithms by utilizing various feature selection techniques. However, it has been less discussed to compare the impact of various feature selection techniques on model performance. This study aims to compare the performance of three different feature selection techniques (filter, wrapper, and evolutionary) in machine learning models for predicting heart disease.

This paper contains the following significant points:

- The present study examines the contributions of different feature selection techniques, filter, wrapper, and evolutionary methods (16 methods) effect on machine–learning algorithms for heart disease prediction.
- In the subsequent phase, all sixteen feature selection techniques were employed with Bayes net, Naïve Bayes (BN), Multivariate Linear Model (MLM), Support Vector Machine (SVM), logit boost, j48, and Random Forest.
- The results were then compared according to the assessment criteria of Precision, F-measure, Specificity, Accuracy, Sensitivity, ROC area, and PRC.
- The most important and significant result of the present study is a comprehensive comparison of a variety of feature selection techniques on machine algorithms for the prediction of heart diseases. The primary and most significant outcome of the study was that, despite the filter methods selecting more features, they were still able to enhance the accuracy factors and precision, as well as F-measures, when applied to machine learning algorithms.
- The most significant improvements in factors are associated with a + 2.3 increase in accuracy after implementation of SVM + CFS/information gain/symmetry uncertainty feature selection methods, as well as an + 2.2 improvement in the F-measure factor derived from SVM + CFS/information gain/symmetry uncertainty.
- The results showed that although feature selection in some algorithms leads to improved performance, in others it reduces the performance of the algorithm.

This paper is structured as follows: Following the introduction in section "introduction", the related literature is reviewed in section "related literature". Research methods are reviewed in section "methodology". The results of the research are presented in section "results". Subsequently, the results of the study are discussed in section "discussion". Finally, the conclusions of the study are presented in section "conclusion". Lastly, the limitations and future scope are discussed in Section "Limitation and future scope".

## Related literature

The Cleveland UCI dataset contains a number of related studies on the prediction of heart disease. These studies fall into two broad categories: the first, which compares algorithms based on classic or deep learning, and the second, which compares the performance of algorithms based on feature selection.

Premsmith et al. presented a model to detect heart disease through Logistic Regression and Neural Network models using data mining techniques in their study. The results demonstrated logistic regression with an accuracy of 91.65%, a precision of 95.45%, a recall of 84%, and F-Measure of 89.36%. This model outperformed the neural network in terms of performance^3^. In a study to enhance heart attack prediction accuracy through ensemble classification techniques, Latha et al. concluded that a maximum of 7% accuracy improvement can be expected from ensemble classification for poor classifiers and those techniques such as bagging and boosting will be effective in increasing the prediction accuracy of poor classifiers^16^. Chaurasia et al. conducted a study to evaluate the accuracy of the detection of heart disease using Naive Bayes (Naive), J48, and bagging. The results indicated that Naive berries provided an accuracy of 82.31%, J48 provided an accuracy of 84.35%, and bagging provided an accuracy of 85.03%. Bagging had a greater predictive power than Naive Bayes^19^.

Mienye et al. presented a deep learning strategy for predicting heart disease in a study utilizing a Particle Swarm Optimization Stacked Semiconductor Auto encoder (SSAE). This research proposes an approach for predicting heart diseases through the use of a stacked SSAE auto encoder that has a softmax layer. The softmax layer is a layer in which the last hidden layer of a sparse Auto encoder is connected to a softmax classifier, resulting in the formation of a SSAE network. This network is then refined with the implementation of the PSO algorithm, resulting in the development of feature learning and enhanced classification capabilities. The application of these algorithms to the Cleveland test yielded the following results: 0.961 accuracy, 0.930 precision, 0.988 sensitivity, and 0.958 F-measure^2^.

In a research project to assess the predictive power of MLP and PSO algorithms for the prediction of cardiac disease, Batainh et al. proposed an algorithm with an accuracy of 0.846 percent, an AUC of 0.848 percent, a precision of 0.808 percent, a recall of 0.883 percent, and an F1 score of 0.844. This algorithm outperforms other algorithms such as Gaussian NB classifiers, Logistic regression classifiers, Decision tree classifiers, Random forest classifiers, Gradient boosting classifiers, K-nearest neighbors classifiers, XGB classifiers, Extra trees classifiers, and Support vector classifiers, and can be used to provide clinicians with improved accuracy and speed in the prediction of heart disease^5^.

In order to enhance the predictive accuracy of heart disease, Thiyagaraj employed SVM, PSO, and a rough set algorithm in a study. To reduce the redundancy of data and enhance the integrity of the data, data was normalized using Z-score. The optimal set was then selected using PSO and the rough set. Finally, the radial basis function-transductive support vector machines (RBF) classifier was employed for the prediction. The proposed algorithm was found to have superior performance compared to other algorithms^7^.

A battery of papers focused on the use of classification techniques in the field of cardiovascular disease. These studies employed classification methods to prognosis the onset of disease, to classify patients, and to model cardiovascular data. The classification and regression tree algorithm (CART), a supervised algorithm, was employed in the studies conducted by Ozcan and Peker to prognosis the onset of heart disease and classify the determinants of the disease. The tree rules extracted from this study offer cardiologists a valuable resource to make informed decisions without the need for additional expertise in this area. The outcomes of this research will not only enable cardiologists to make faster and more accurate diagnoses but will also assist patients in reducing costs and improving the duration of treatment. In this study, based on data from 1190 cardiac patients, ST slope and Old peak were found to be significant predictors of heart disease^15^.

Bhatt et al., in their study based on data from Kaggle datasets and using Random Forest, Decision Tree Algorithms, Multilayer Perception, and XGBOOST classifier, predicted heart disease. In conclusion, the MLP algorithm demonstrated the highest level of accuracy (87.28%) among the other algorithms evaluated^14^. In a study conducted by Khan et al., 518 patients enrolled in two care facilities in Pakistan were predicted to develop heart disease using decision tree (DT), random forest (RF), logistic regression (LR), Naive Bayes (NB), and support algorithms. The most accurate algorithm used to classify heart disease was the Random Forest algorithm, which had an accuracy of 85.01%^20^. This was the best out of the other algorithms, according to a study by Kadhim and colleagues. They looked at a dataset of IEEE-data-port data sources and used a bunch of different algorithms to classify it. The Random Forest algorithm was the most accurate, with an accuracy of 95.4%^21^. In addition to these papers, a further set of studies have explored the application of machine learning to image and signal analysis.

Medical images are a critical tool in the diagnosis of a variety of medical conditions, including tumors. Due to the high degree of similarity between radiological images, timely diagnosis may be delayed. Consequently, the utilization of machine learning techniques can lead to an increase in the rate and precision of medical image-based diagnosis. Furthermore, with the growing number and volume of medical images available, the search for similar images and patients with similar complications can further enhance the speed and precision of diagnosis. The WSSENET (weakly supervised similarity assessment network) was a method used to evaluate the similarity of pulmonary radiology images, and it was found to be more accurate in retrieving similar images than prior methods^22^. In this paper^23^, a low-dose CT reconstruction method is proposed, based on prior sparse transform images, to resolve image issues. This method involves the learning of texture structure features in CT images from various datasets, and the generation of noise CT image sets to identify noise artifact features in CT images. The low-dose CT images processed with the enhanced algorithm are also used as prior images to develop a novel iterative reconstruction approach. DPRS is a method employed to expedite the retrieval of medical images within telemedicine systems, resulting in an enhanced response time and precision. Classification and selection of features are also employed for medical photo classification. Deep learning was employed to classify medical images in the study^24^. The adaptive guided bilateral filter was employed to filter the images. In this study, Black Widow Optimization was also employed to select the optimal features. The accuracy rate achieved in this study was 98.8% when Red Deer Optimization was applied to a Gated Deep Relevance Learning network for classification. Metaheuristic approaches have gained increased recognition in the scientific community due to their reduced processing time, robustness, and adaptability ^25^ . In his study presented a methodology based on a multi-objective symbiotic organism search to solve multidimensional problems. The results of a Feasibility Test and Friedman's Rank Test demonstrated that this method is sufficiently effective in solving complex multidimensional problems with multiple axes. A triangular matching algorithm was used in the study^26^. The method of soft tissue surface feature tracking is presented in the study. A comparison of the results of the soft tissue feature tracking method with the results of the convolution neural network was conducted. The result showed that the method of soft tissue feature tracking has a higher degree of accuracy. In a study (Dang et al.), a matching method was presented to overcome the issues of conventional feature matching. The method of matching feature points in various endoscopic video frames was presented as a category, and the corresponding feature points in subsequent frames were compared with the network classifier. The experimental data demonstrated that the feature-matching algorithm based on a convolutional network is efficient due to feature-matching, no rotation displacement, and no scaling displacement. For the initial 200 frames of a video, the matching accuracy reached 90%^27^. In a study, Ganesh et al. used a wrapper method based on the K Nearest Neighborhood (KNN) algorithm to select the best features. In this study, the WSA algorithm was compared with seven metaheuristic algorithms. The results showed that this algorithm was able to reduce 99% of the features in very large datasets without reducing the accuracy and performed 18% better than classical algorithms and 9% better than ensemble algorithms^28^ . Priyadarshini et al. conducted a study using metaheuristic algorithms inspired by physics investigated feature selection. The performance of these algorithms were compared using factors such as accuracy, processing cost, suitability, average of selected features and convergence capabilities. The results showed that Equilibrium Optimizer (EO) had a better performance than other algorithms and it was suggested to solve problems related to feature selection^29^.

The following is a summary of the findings of the studies comparing the feature selection techniques and the algorithms used in the Cleveland dataset to predict heart diseases (Table1).

**Table 1:** Related studies with a focus on feature selection effect on heart disease prediction.

Feature selection method	Classification algorithm	Evaluation factor	Year	References
Chi-squared and analysis of variance (ANOVA)	Logistic regression, k-nearest neighbor, decision tree, random forest, Gaussian naive bayes, extra gradient boosting, support vector classifier, multilayer perceptron, stochastic gradient descendent, and additional tree classifier	Accuracy	2023	^18^
Meta-heuristic algorithms(CS, FPA, WOA, and HHO)	SVM, KNN, Random Forest, Naïve Base, Logistic Regression	F-score and AUC	2023	^8^
Relief, Info gain Chi-squared Filtered subset One attribute based Consistency based Gain ratio Filtered attribute CFS, Genetic algorithm	Multilayer perceptron, KNN, SVM, J48	Accuracy	2019	^30^
Fast Correlation-Based Feature Selection (FCBF), PSO and ACO	KNN, SVM, RF, NB, MLP	Accuracy	2018	^1^

This group of studies included only a few feature selection techniques mostly filter methods as well as accuracy factor, as indicated in Table 1. However, in this study, sixteen feature selection methods in three groups filters, wrapper, and evolutionary were studied and their impact on all factors-including Precision, F-measure, Specificity, Accuracy, Sensitivity, ROC area, and PRC were measured.

## Methodology

**Figure 1:**
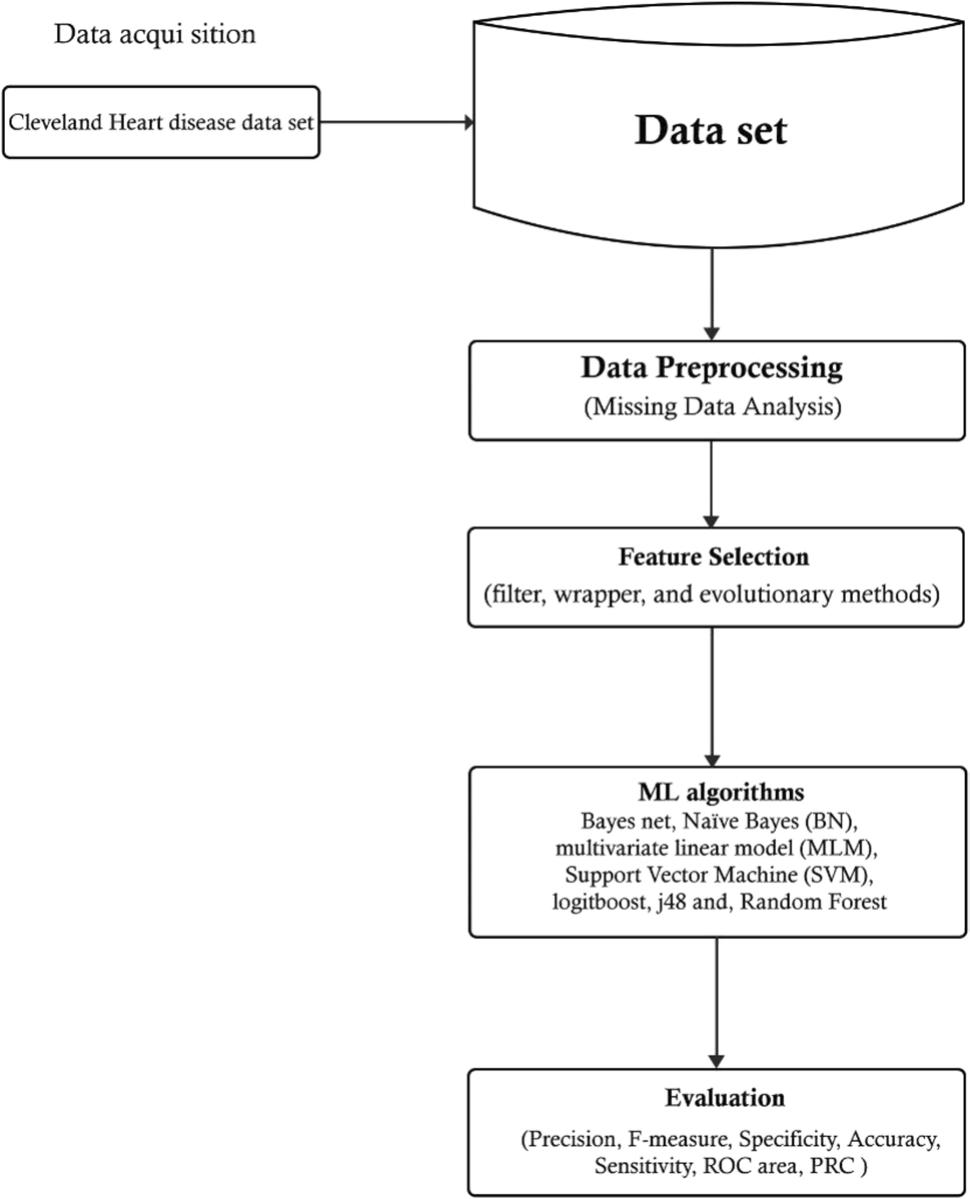
Study phases.

The present study was divided into four general phases, as illustrated in Fig. 1.

Once the data had been acquired and preprocessed, sixteen feature selection techniques were applied in three categories: filter, wrapper, and evolutionary methods. Subsequently, the best subset was selected, and seven machine-learning techniques applied. Subsequently, algorithm and feature selection performance were evaluated using various evaluation factors. Since a public dataset was used in this study, informed consent was not obtained. In addition, human subjects were not used in present research. Also, all stages of the research were in accordance with the standards and guidelines of ethics in research, and the study was conducted after obtaining the code of ethics in the ethics board of Shiraz University of Medical Sciences.

### Dataset

The dataset used for the heart disease analysis is the Cleveland Heart disease dataset. This dataset was extracted from UCI Machine Learning Repository and consists of 303 records. This dataset includes a total of 165 individuals with cardiovascular disease and 138 individuals with no cardiovascular case history. The dataset was characterized by 13 attributes for predicting heart disease, with one attribute serving as the final endpoint. Table 2 provides a description of this dataset.

**Table 2:** The detail of Cleveland dataset.

Attributes	Explanation	Type	Value
Age	Age in years	Numeric	29–77
Sex	gender	Binary	Male = 1, Female = 0
Cp	Chest pain type	Nominal	1 = typical angina, 2 = atypical angina, 3 = non-anginal pain, 4 = asymptomatic
Trestbps	Resting blood pressure in mmHg	Numerical	94–200
Chol	Serum cholesterol in mg/dl	Numeric	126–564
FBS	Fasting blood sugar > 120 mg/dl	Binary	True = 1, False = 0
Restecg	Resting electrocardiographic results	Binary	Normal = 0, Abnormality = 1
Exang	Maximum heart rate achieved	Numeric	71–202
Oldpeak	ST depression induced by exercise relative to rest	Numeric	0–6.2
Slope	The slope of the peak exercise ST segment	Nominal	1 = upsloping, 2 = flat, 3 = down sloping
Ca	Number of major vessels colored by fluoroscopy	Numeric	0–4
Thal	Defect type	Nominal	3 = Normal. 6 = Fixed defect 7 = reversible defect
Target	Healthy or patient	Binary	1 = healthy, 0 = patient

Data preprocessing is one of the most critical steps after obtaining the data. Due to the uniformity and global nature of the data set, only the missing value analysis was used as a pre-processing technique, and records with blank fields were eliminated from the data set. At this stage, the dataset has been filtered for missing data and 6 missing records were removed, leaving 297 records to be processed.

### Feature selection

Feature selection is the process of removing unrelated and repetitive features from a dataset based on an evaluation index to make it more accurate. There are three main types of feature selection methods: filter, wrapper, and embedded^31^. Filtering methods use the general properties of the training data to perform the selection as a step-by-step process independent of an induction algorithm. Filtering methods have lower computational complexity and are better at generalizing. Because filter methods only look at the intrinsic properties of the training samples to evaluate a feature or a group of features, they can be used with a wide range of classifiers^32^.

In a wrapper-based method, the selection process involves optimization of a predictor. Unlike a filter method, a wrapper method is tailored to a particular classifier and evaluates the quality of a subset of candidates. As a result, a wrapper method achieves better classification performance than a filter method. In a third-party method, feature selection is performed during the training phase. Embedded methods constitute a subset of overlay methods, which are characterized by a more profound relationship between feature selection and the classifier construction^33^. Feature subsets are formed when the embedded methods are used to construct the classifier^32,33^.

In the present study, filter methods were employed alongside wrapper and evolutionary methods (Fitness function: precision + SVM), which are briefly outlined below.

#### Filter method

*Correlation-based feature selection (CFS): *This multivariate filter algorithm ranks feature subsets based on a heuristic evaluation function based on a correlation. The bias function evaluates subsets that correlate with the class and are not correlated with other features. Non-relevant features are disregarded as they will not have a high correlation with the class; additional features should be evaluated as they are highly correlated to one or more other features. The acceptance of a feature is dependent on its ability to predict classes in areas of the sample space that have not previously been predicted by other features^32^.

*Information gain*: This univariate filter is a widely used way of evaluating features. It assigns an order of importance to all features and then determines the necessary threshold value. In this example, the threshold value is determined by selecting features that receive positive information gain^32^.

*Gain ratio*: The purpose of the algorithm modified for information gain is to mitigate bias. The algorithm evaluates the number and scope of branches when selecting a feature. By taking into account the internal information of a segment, the algorithm attempts to adjust the information gain^34^.

*Relief*: This method involves selecting a random sample of data and then finding the closest neighbor of that class and its counterpart. The closest neighbor’s attribute values are then compared to the sample and the associated scores for each attribute are updated. The logic for each attribute is that it distinguishes between samples from different classes and takes the same value into account for samples that belong to the same class^32^.

*Symmetrical uncertainty*: To determine the relationship between a feature and a class label, symmetric uncertainty is used. The mean normalized mutual benefit of a feature (f), each other feature (n), and the class label reflects the relationship between feature f and other features in a set of features (F)^35^.

#### Wrapper method

*Forward and backward selection*: In a backward elimination model, all features are eliminated and the least important features are removed sequentially. In a forward selection model, no features are eliminated, and the most important features are added sequentially^36^.

*Naïve Bayes*: This algorithm is derived from probability theory to identify the most likely classifications. It utilizes the Bayes formula (Eq. 1) to determine the likelihood of a data record Y having a class label c_j_^11^.

1 $$P(label = c_{j} |Y) = \frac{{P(Y|label = c_{j} )*P(c_{j} )}}{P(Y)}$$

*Decision tree*: The tree-based technique involves each path beginning at the root of the tree is initiated by a sequence of data separators, and the sequence continues until the result reaches the leaf node. The tree-based technique is, in reality, a hierarchy of knowledge that consists of nodes and connections. Nodes, when used for classification purposes, represent targets^37^.

*K-Nearest-Neighbor (KNN)*: It is a classifier and regression model used for classification. As KNNs are typically sample-based (or memory-based) learning schemes, all computational steps in KNNs are postponed until classification. Furthermore, KNNs do not require an explicit training step to construct a classifier^33^.

*NN*: A neural network is a computer model composed of a vast number of interconnected nodes, each of which represents a particular output function, referred to as an activation function. Each node represents a signal, referred to as a weight that passes through the connection between two nodes. The weight corresponds to the memory capacity of the neural network, and the output of the neural network will vary depending on how the nodes are connected, the degree of weight, and the incentive function^38^.

*SVM*: Support vector machines (SVM) are algorithmic extensions of statistical learning theory models that are designed to generate inferences that are consistent with the data. The question of estimating model performance in an unfamiliar data set, taking into account the model’s properties and the model’s performance in the training set is posed by support vector machines. These machines solve a restricted quadratic optimization problem to find the optimal dividing line between sets. The model generates data, and different kernel functions can be employed to provide varying degrees of linearity and flexibility^39^.

*Logistic regression*: Logistic regression (or logistic regression analysis) is a statistical technique that involves the prediction of the outcome of a class-dependent variable (or class of variables) from a set of predicted variables. Logistic regression involves the use of a binary dependent variable (or class) with two categories and is primarily used to predict, as well as to calculate, the probability of a given outcome^40^.

#### Evolutionary algorithms

They are a type of metaheuristic algorithm based on population that involves the use of a set of solutions in each step of the solution process. This set of solutions is composed of operators that combine/change solutions to incrementally improve/evolve aggregate solutions based on the Proportion uses function. This category includes algorithms such as PSO, ABC, and genetic algorithms^41^.

*Artificial Bee Colony (ABC)*: ABC is a hybrid population-based optimization algorithm in which artificial bees act as change operators to refine the solutions to the optimization problem-i-e-of food resources. The objective of the bees is to locate food sources with the primary nectar. In ABC, an artificial bee navigates a multidimensional area and selects nectar resources based on experience and hive companions or based on its location. In addition, some bees fly (explore) and select food sources randomly, without relying on experience. When they locate a source of the primary nectar, they retain their positions. ABC combines local and global search methods to achieve a balance between exploration and utilization of the search space^42^.

*Genetic algorithm*: A genetic algorithm is a type of programming technique that utilizes evolutionary biology techniques, including heredity, mutation, and the principles of Darwin’s selection, to find the most appropriate formula to predict or match a pattern. In many cases, genetic algorithms are a suitable substitute for regression-based prediction methods. Genetic algorithm modeling is a programming approach that utilizes genetic evolution as a tool for problem-solving. Inputs are transformed into solutions through a process model based on genetic evolution, and the solutions are then evaluated as candidates for the fitness function. If the output condition of the problem can be met, the algorithm is terminated. In general; a genetic algorithm is an algorithm that is based on repetition, with most of its parts selected as random processes. It consists of parts of a function of fitting, displaying, selection, and change^43^.

*Particle swarm optimization (PSO): *In particle swarm optimization algorithms, each member of a population or solution is referred to as a particle. Each particle flies and moves through the search space with its initial position and velocity to locate the most optimal solution. Each particle stores the best position it has achieved while searching and moving through the search space as its own experience. This information is then shared with other particles within the neighborhood, allowing them to identify the locations where they had the greatest success and thus the best position within their neighborhood or the entire search space. The best group experience is known as the solution^1^.

### Machine learning algorithms:

This study employed a variety of machine learning models, including Bayes net, Naïve Bayes (BN), multivariate linear model (MLM), Support Vector Machine (SVM), logit boost, j48, and Random Forest. Bayes nets are mathematical models that represent relationships among random variables through conditional probabilities, similar to how a classifier evaluates the probability of P(c| x) of a class of discrete variables c in the presence of certain characteristics of a given X pay^44^. Random forests are a subset of tree based models, in which tree predictors are calculated independently from a random vector’s values after a distribution that is equivalent for all trees within the forest. The generalization error of random forest classifiers is contingent upon the relationship between individual trees in the forest and the strength of those trees. J48 classifiers are extensions of the classification decision tree algorithm (C4.5) that generate binary trees. This system constructs a tree to represent the classification procedure. After constructing the tree, the algorithm applies to any tuple within the database to classify that tuple^45^.

An MLP is a supervised learning approach that utilizes back-propagating techniques. Because there are many layers of neurons in an MLP, it can be considered a deep learning approach and is commonly employed to solve supervised learning problems. Additionally, it has been used in computational neuroscience research as well as in distributed parallel processing (DCP) research^46^. The logit boost is a boosted classification algorithm that is based on incremental Logistic regression and strives to reduce logistic loss.

### Evaluation and analysis tools:

For data analysis and the identification of significant risk factors, Waikato environment for knowledge analysis (Weka) version 3.3.4 was utilized. Evolutionary algorithms were implemented in Matlab 2019b, and machine learning models were implemented in R 3.4.0. The models were validated using a tenfold cross-validation method and various criteria, such as accuracy, sensitivity, specificity, and precision, as well as F-measure, ROC, and PRC area (Table 3). These indices operate based on the confusion matrix, a two-dimensional matrix that compares the predicted class values to the actual class values. Within the first quartile, true positives (TP) refer to the number of correctly classified patients with heart disease, and false positives (FP) refer to patients without heart disease who are incorrectly classified as having heart disease. The False Negative (FT) refers to patients with heart disease that are not classified correctly by the model, while TN (true negative) refers to patients without heart disease that are classified correctly^12^. The f-measure, the ROC area, and the PRC area indices are aggregated indices that provide an overall assessment of the model; the mathematical formulas (Eqs. 2–6) for the calculation of the assessment indices are outlined in Table 3.

**Table 3:** Study performance indices.

Performance criteria	Calculation
Accuracy	$\frac{TP + TN}{{TP + FP + TN + FN}}$(2)
Precision	$\frac{TP}{{TP + FP}}$(3)
Sensitivity/Recall	$\frac{TP}{{TP + FN}}$(4)
Specificity	$\frac{TN}{{FP + TN}}$(5)
F-score	$2*\left( {\frac{{{\text{Precision}}*{\text{Sensitivity}}}}{{{\text{Precision}} + {\text{Sensitivity}}}}} \right)$(6)

### Ethical statement

The study protocol was approved by the Shiraz University of Medical Sciences (SUMS) Ethics Board. Approval Date: 2022-11-19; Approval ID: IR.SUMS.NUMIMG.REC.1401.097.

## Results

The heart disease dataset consisted of 297 records (after removing 6 missing records) in which 160 subjects (53.9%) had no heart disease, and 137 subjects (46.1%) had heart disease. To determine the risk factors associated with heart disease diagnosis, sixteen feature selection methods were applied in three categories: filter, wrapper, and evolution. All of the feature selection techniques were employed on the features, and the outputs of each operation, as well as the features chosen by each technique, are presented in Table 4.

**Table 4:** Feature selection results.

Type of algorithm	Feature selection technique	No selected features	Age	Sex	Cp	Trestbps	Chol	FBS	Restecg	Thalach	Exang	Oldpeak	Slope	Ca	Thal
Filter	CFS	10	*	*	*				*	*	*	*	*	*	*
	Information Gain	10	*	*	*				*	*	*	*	*	*	*
	Gain ratio	11	*	*	*			*	*	*	*	*	*	*	*
	Relief	12	*	*	*	*		*	*	*	*	*	*	*	*
	Symmetrical uncertainty	10	*	*	*				*	*	*	*	*	*	*
Wrapper	Forward selection	5			*						*	*		*	*
	Backward selection	4			*							*		*	*
	SVM	9	*	*	*		*	*			*	*	*	*	
	NB	6		*	*					*	*	*		*	
	Logistic regression	9	*	*	*		*		*			*	*	*	*
	NN	11		*	*	*		*	*	*	*	*	*	*	*
	KNN	7		*	*			*	*			*		*	*
	Decision tree	7		*	*			*	*			*		*	*
Evolutionary (Fitness function: accuracy + SVM)	Genetic algorithm	9	*	*	*	*	*			*			*	*	*
	PSO	7			*				*	*	*	*		*	*
	Artificial Bee Colony	7	*				*	*	*	*			*	*	

*Selected features are shown with star mark (*), Cp: chest pain, FBS: fasting blood sugar, restECG: rest electrocardiographic, Exang: exercise-induced angina, Slope: peak exercise slope measure, Ca: number of major vessels colored by fluoroscopy, Thal: heart rate, Trestbps: resting blood pressures of patients measured in mm Hg on admission to the hospital, Chol: serum cholesterol, Thalach: maximum heart rate, Old peak: ST depression made by exercise relative to rest.

The results of Table 4 demonstrate that the forward and backward regression methods have selected the minimum number of features, while the Relief method has selected the most (n = 12). In the subsequent step, seven different machine-learning methods were employed. The performance of these methods was evaluated using the tenfold cross-validation technique. All models were initially implemented based on the complete data set, followed by the features selected by the feature selection methods.

After implementing all feature selection methods and determining the number of selected features, the results of Table 5 show the methods that selected the least number of features in each category. According to this, wrapper algorithms choose the least features while filter methods choose the most features. In addition, all the features selected by the filter algorithms were similar, while evolutionary algorithms, despite the same number of features, chose different feature types.

**Table 5:** Minimum features which choose by different methods.

Methods	Feature selection algorithm	Number of selected features	Selected features
Filter	CSF	10	age, sex, cp, restecg, thalach, exang, oldpeak, ca, thal, slope
	Information Gain	10	age, sex, cp, restecg, thalach, exang, oldpeak, ca, thal, slope
	Symmetrical uncertainty	10	age, sex, cp, restecg, thalach, exang, oldpeak, ca, thal, slope
Wrapper	Backward selection	4	cp, oldpeak, ca, thal
	Forward selection	5	exang, cp, oldpeak, ca, thal
Evolutionary	PSO	7	cp, resteg, thalach, exang, oldpeak, ca, thal
	ABC	7	age, chol, fbs, resteg, thalach, slope, ca

**Figure 2:**
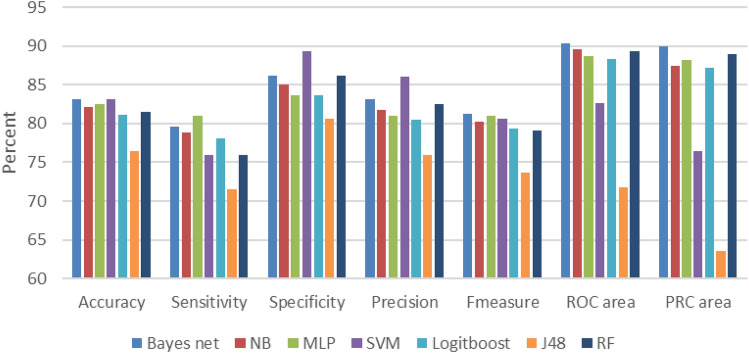
Before FS (based on the original data set).

The results of running the machine learning algorithms before feature selection are presented in Fig. 2.

The SVM algorithm achieves a good performance with ACC = 83.165%, Spec = 89.4%, and Precision = 86. However, when the combined criteria are taken into account, Bayesian networks achieve better performance with ACC = 81.3%, F = 81.3%, AUC = 90.3%, and PRC = 90. The highest sensitivity value achieved for MLP was 81%.

**Figure 3:**
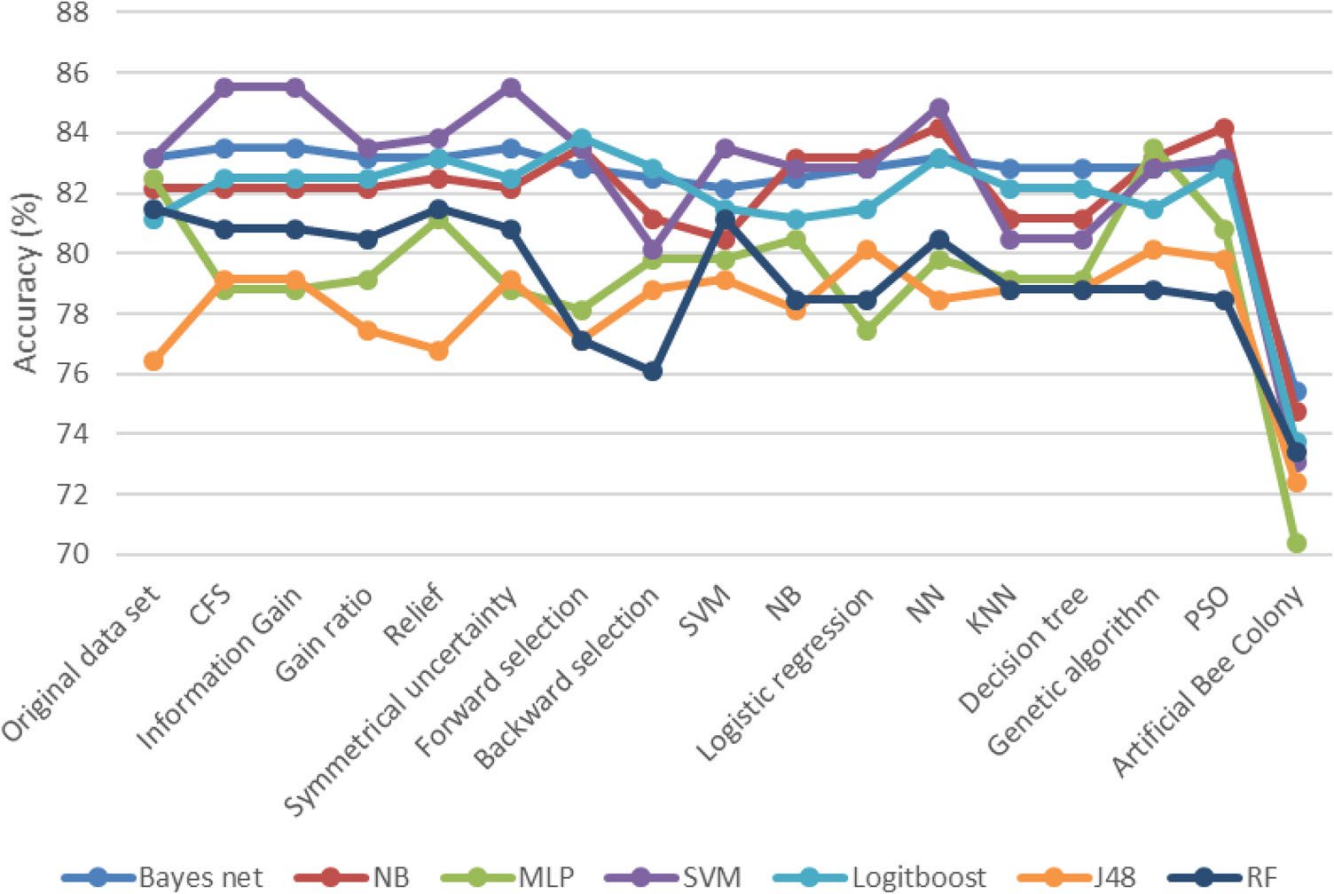
Accuracy result of the algorithm after feature selection.

**Figure 4:**
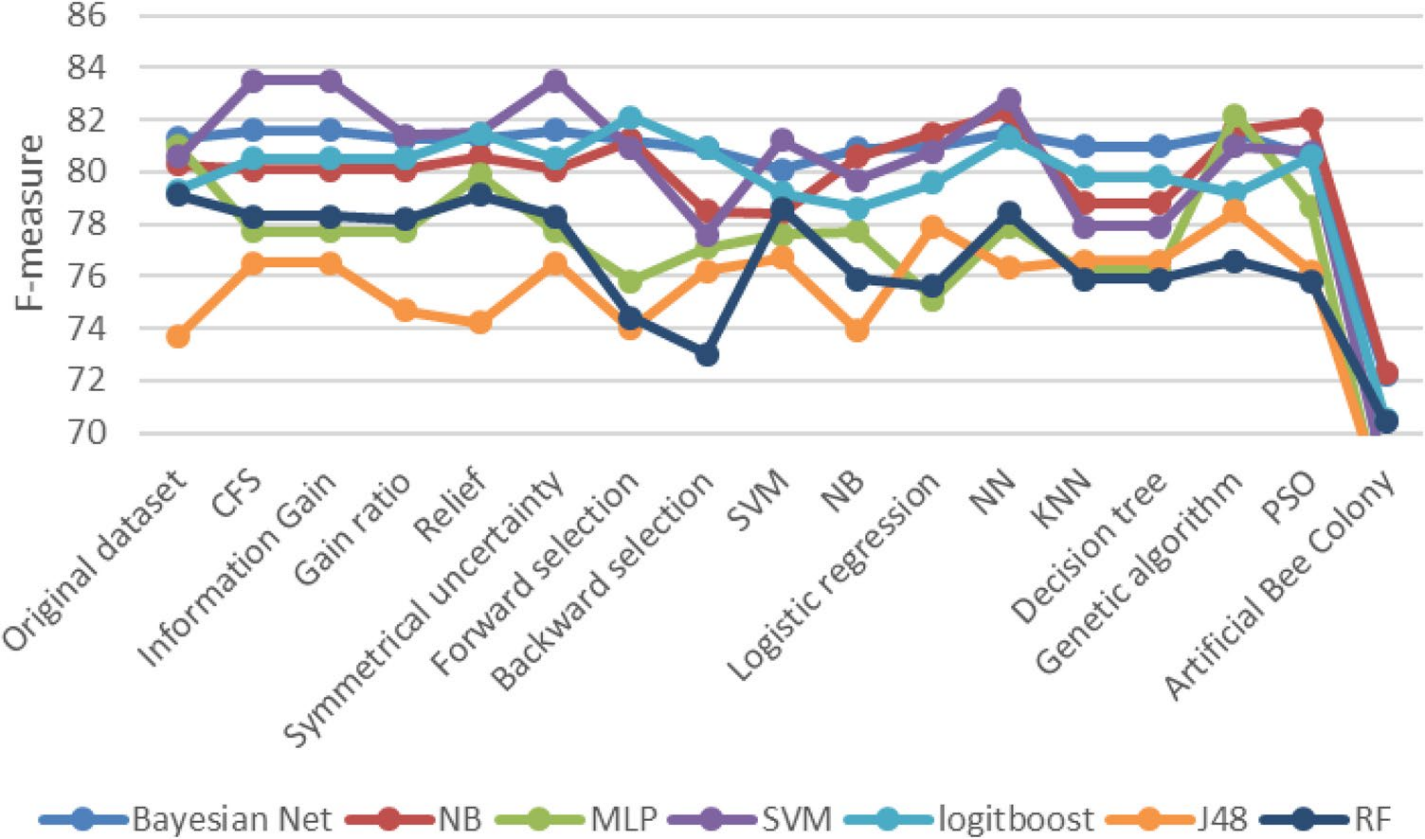
F-measure results after Feature selection.

**Figure 5:**
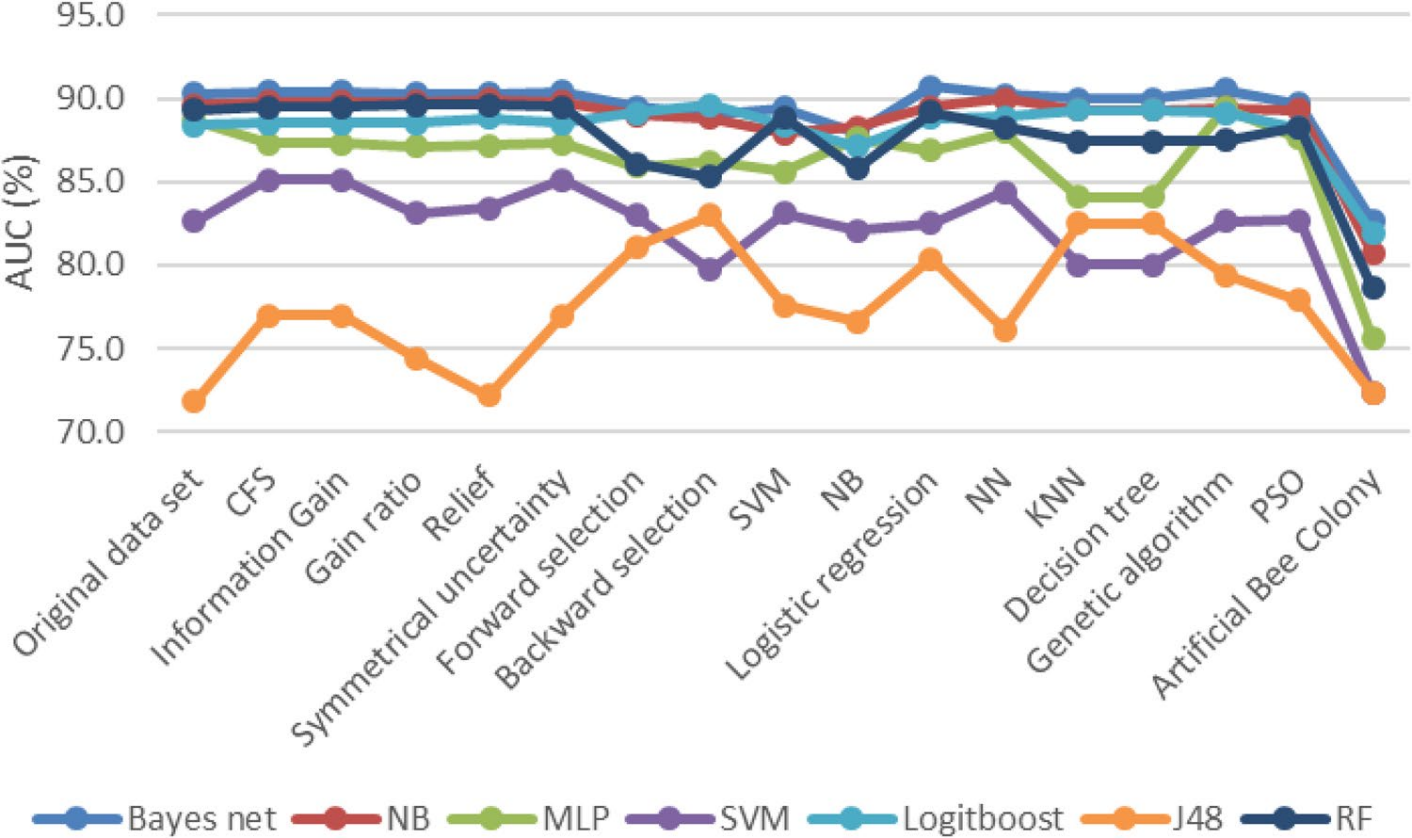
AUC results after Feature selection.

Figures 3, 4 and 5 compare the machine learning algorithms' performance after feature selection for accuracy, F-measure, and ROC diagram area.

The accuracy of all algorithms is demonstrated in Fig. 3 following the selection of features. The SVM algorithm implemented using the CFS/Information Gain/Symmetrical Uncertainty feature selection method displays the highest performance in comparison to other algorithms. The Bayes net algorithm displays the highest performance after the implementation of feature selection methods.

The values associated with the F-measure are presented in Fig. 4 following the implementation of the algorithms based on the feature selection methods. The highest performance was associated with the SVM + CFS/information gain / Symmetrical uncertainty algorithm.

In Fig. 5, the AUC values are displayed after performing the feature selection methods. Bayesian Network + Wrapper-logistic Regression algorithm had the best performance among other algorithms. As can be seen in the picture, the amount of AUC has been improved after feature selection in most algorithms.

The results demonstrate that feature selection resulted in significant improvements in model performance in some methods (e.g., j48), while it led to a decrease in model performance in other models (e.g. MLP, RF). Table 6 compares the best results achieved before and after feature selection.

**Table 6:** Performance result comparison before and after feature selection.

Performance metric	Before FS		After FS		Differences
	Best value	ML technique(s)	Best value	ML technique + FS algorithm(s)	
Accuracy	83.2	SVM, Bayesian Network	85.5	SVM + CFS/information gain/Symmetrical uncertainty	+ 2.3
Sensitivity	81	MLP	82.5	MLP + GA	+ 1.5
Specificity	89.4	SVM	91.2	SVM + Wrapper-NB	+ 1.8
Precision	86	SVM	87.9	SVM + CFS/information gain/Symmetrical uncertainty	+ 1.9
F-measure	81.3	Bayesian Network	83.5	SVM + CFS/information gain/Symmetrical uncertainty	+ 2.2
ROC area	90.3	Bayesian Network	90.7	Bayesian Network + Wrapper- Logistic Regression	+ 0.4
PRC area	90	Bayesian Network	90.2	Bayesian Network + Wrapper- NN	+ 0.2

Table 6 demonstrates that filter feature selection techniques have improved model performance in terms of Accuracy, Precision, and F-Measure, however, Wrapper-based and evolutionary algorithms have enhanced model sensitivity and specificity. SVM-based filtering methods have a best-fit accuracy of 85.5. In fact, in a best-case scenario, filtering methods result in + 2.3 model accuracy. SVM-based feature selection methods have the highest improvement in this index, with the PRC index having the lowest improvement of + 0.2.

**Figure 6:**
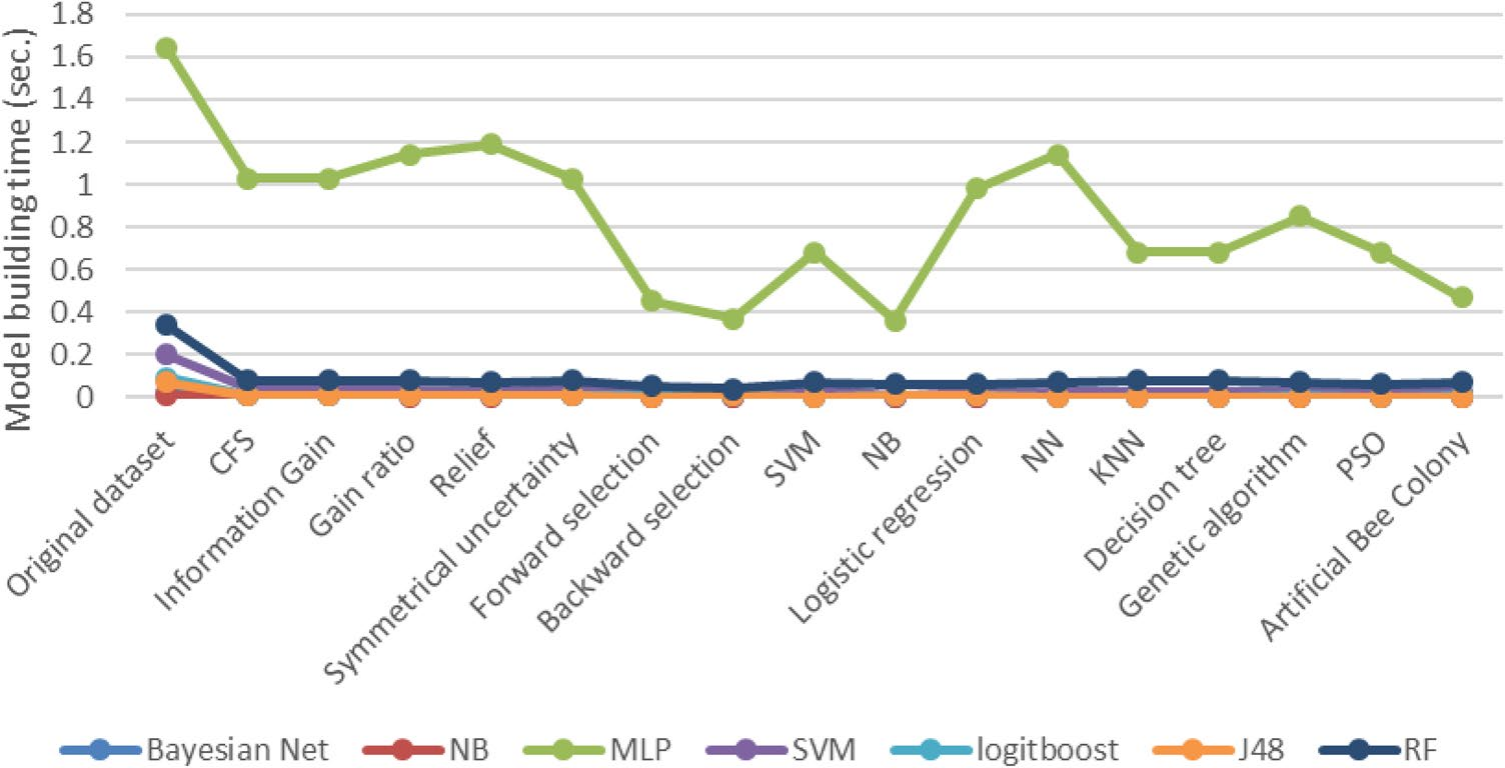
ML models ‘execution time before and after feature selection.

Figure 6 shows the ML model running time before and after the feature selection. All models are running on Corei3 (RAM = 4GB). The comparison of the results shows that the ML models with the original set of data reached an average model building time of 0.59 ± 0.34 s, among which MLP with 1.64 s and NB with 0.01 s had the highest and lowest times. Following the implementation of the feature selection methods, the ML models with the features selected by the Relief and gain ratio method achieved an average model building time (ABT) of 0.44 ± 0.19 s and 0.42 ± 0.18 s respectively. Additionally, the backward method and the Wrapper + NB method resulted in an ABT of 0.14 ± 0.06 and an average model construction time of 0.13 ± 0.06, respectively.

Table 6 summarizes the findings of this study and related papers.

Based on the data presented in Table 7, the accuracy of this paper was 85.5% higher than that of similar papers based on the SVM algorithm and the CFS/Information Gain/Symmetrical Uncertainty Feature selection methods.

**Table 7:** Comparative accuracy results of similar studies compared to present study.

Feature selection methods	ML algorithms	Best algorithm performance	Best accuracy (%)	Year	References
Feature reduction (11 features)	Naive Bayes, J48, and bagging	Bagging	85.03	2014	^19^
Chi-squared feature evaluator	Random forest	RF	83.7	2015	^47^
Feature reduction	Naive Bayes, KNN, decision tree, and bagging	KNN	79.2	2017	^48^
Brute force feature selection	Bayes Net, Naive Bayes, Random forest, C4.5, Multilayer perceptron, PART, majority voting	Majority voting	85.48	2019	^16^
linear discriminant analysis (LDA), hybrid feature selection algorithm, and medical doctors’ recommendation-based feature selection	Naive Bayes (NB), Random Forest (RF), k-nearest Neighbor (KNN), support vector machine (SVM), Extreme Gradient Boosting (XGBOOST)	SVM	81.84	2019	^49^
PC features, Chi-squared, Relief-F, symmetrical uncertainty	Bayes Net, Logistic, Stochastic Gradient Descent (SGD), KNN, random forest	Chi-squared feature selection with the Bayes Net algorithm	85.00	2020	^12^
filter methods (CSF, Information Gain, Gain Ratio, Relief, Symmetrical uncertainty), Wrapper (Forward and backward selection, Naïve Bayes, Decision tree, KNN, NN, SVM, Logistic regression), and evolutionary** (**PSO, ABC, and genetic algorithms)	Bayes Net, Naïve Bayes (BN), multivariate linear model (MLM), Support Vector Machine (SVM), logit boost, j48, and Random Forest	SVM + CFS/information gain/Symmetrical uncertainty	85.5	2023	Our study

## Discussion

This study evaluates the influence of filter selection methods on the performance of various algorithms. Firstly, the algorithms were applied to the dataset without the implementation of feature selection methods. The SVM and Bayesian Network algorithms demonstrated the most robust performance, with accuracy values of 83.2 and 83.0 respectively. However, when combined criteria such as the F-measure = 81.3, the AUC = 90.3, and the PRC area = 90, the Bayesian network performed more efficiently. Subsequently, sixteen feature selection methods were applied in three categories: the filter, the wrapper, and the evolutionary. The wrapper method selected the least number (backward selection = 4, forward selection = 5) and the filter method selected the most features (Relife = 12). Evolutionary methods PSO and ABC also selected 7 features. Although the numbers were similar, the selection of features varied between the two algorithms. In his analysis of feature selection correlation methods for predicting heart disease, Reddy concluded that the highest level of accuracy could be achieved with the selection of 8 features, however, when the number of features was reduced to 6, no improvement in performance was observed. Conversely, when the selection method was changed and only 3 features were selected, an increase in accuracy was observed. He concluded that the selection method and type of features selected can have a significant impact on the algorithms' performance^50^. The highest accuracy in this study is associated with SVM = 85.5 using the CFS/Information Gain/Symmetrical Uncertainty Filter method after the three feature selection methods were applied. All three methods selected the same ten features. While filter methods resulted in more features selected than other wrapper or evolutionary methods, they also generated greater accuracy. In Gokulnath's study, the filtering methods were found to have increased the model's accuracy, with filter methods yielding the most significant improvement in the F-measure index (2.2)^30^. In his study on cardiovascular disease, Şevket Ay concluded that the selection of the feature through metaheuristics such as cuckoo search (CS), flower pollination algorithm (FPA), whale optimization algorithm (WOA), and Harris hawks (HH) resulted in an improvement in the F-score and the AUC indices^8^. In the present study, the genetic algorithm was only able to increase the sensitivity index by 1.5 compared to other methods. The wrapper-based feature selection methods were found to improve the ROC area and PRC area, as well as the specificity indicators. Furthermore, the results of the study indicated that feature selection does not always result in model improvement. For instance, two algorithms (MLP and RF) experienced a decrease in performance following feature selection.

The present study was able to achieve a higher performance in terms of accuracy by achieving a performance of 85.5% compared to other similar papers (Table 7). This result was obtained after implementing CFS/information gain/Symmetrical uncertainty feature selection methods. The results of the present study showed that feature selection can lead to the improvement of most of the evaluation indices of the algorithms in the prediction of heart disease, and the highest improvement was observed in the accuracy index. All 16 feature selection methods were implemented with all algorithms, resulting in new insights into feature selection methods. Indeed, one of the key findings of this study was the influence of various feature selection groups on algorithm performance. Also, the results of the present study showed that the feature selection methods that lead to the selection of the least number of features cannot achieve the best improvement in the model's performance. The results of our study indicated that the best accuracy was obtained based on filter methods that selected more features than other methods. These results can contribute to the issue that maybe the type of variables has a greater impact on building the model than the number of selected features. Also, every feature selection method may not lead to improved model performance. Therefore, comparing various feature selection methods and measuring their impact plays a significant role in building the best prediction model.

## Conclusion

Artificial Intelligence (AI) technologies have advanced to a point where they offer deep, efficient, and non-intrusive analytical capabilities to facilitate the decision-making of physicians and health policy-makers in comparison to conventional methods^9,10^. In addition, the utilization of Machine Learning (ML) models in support of medical diagnoses, screening, and clinical prognosis is on the rise due to their high capacity to identify and categorize patients^51^. Presently, clinical professionals are confronted with a vast amount of health data that is both complex and imprecise, making it difficult for them to make informed decisions^52^. The speed of decision-making in heart diseases has the potential to reduce complications and improve the patient's condition, whereas machine learning algorithms have been instrumental in predicting, diagnosing, and treating various diseases through their high accuracy.

The main findings of this study are as follows:

- This study examines the role of feature selection methods in optimizing machine learning algorithms in heart disease prediction.
- Based on the findings, the filter feature selection method with the highest number of features selected outperformed other methods in terms of models' ACC, Precision, and F-measures.
- Wrapper-based and evolutionary algorithms improved models' performance from a sensitivity and specificity point of view.
- Based on current knowledge, this study is among the few to compare the performance of different feature selection methods against each other in the heart disease algorithm field.
- Previous research has mainly focused on enhancing algorithms, whereas studies that have examined the impact of feature selection on the field of cardiac prediction have focused on a limited number of methods, such as filter or metaheuristic.
- As a result, the findings of this study may be of value to health decision-makers, clinical specialists, and researchers. The findings of this study will enable clinical professionals to utilize artificial intelligence more effectively in the prediction of heart disease. Policymakers will be able to plan and allocate resources for the utilization of AI in the area of health promotion and prevention of cardiovascular disease, and researchers can draw on the findings of this study to inform further research on the function of feature selection methods across various fields of disease.

## Limitation and future scope

The limitations of this study include the use of a single dataset and the utilization of only seven algorithms. It appears that improved results can be obtained by utilizing multiple datasets and additional algorithms. Another limitation of this study is that socio-economic characteristics and other clinical characteristics related to people’s lifestyle (e.g., smoking, physical activity) were not taken into account. Future studies will be able to provide better results by taking into account a broader range of clinical characteristics and socio-economic characteristics. However, other information (e.g. patient medical images, ECG signals) were not included in this study. The simultaneous utilization of structured and non-structured data, signals, and medical images, can provide researchers with more comprehensive insights and thus serve as a foundation for future exploration. Furthermore, the limited size of the dataset studied may limit the ability to disseminate the findings of the current study to the general public, thus necessitating the utilization of larger datasets and larger sample sizes to enhance the outcome of future research. Therefore, based on the findings of this paper, the present research team will focus on using larger datasets with a wider range of features and will also look at the impact of different feature selection techniques on different disease domains and, finally, the current team will employ more algorithms and, of course, deep learning techniques.

## Data Availability

The public repository UCI Machine Learning Repository, Cleveland Heart disease data set were used for analyzing which is public and can retrieve from https://archive.ics.uci.edu/ml/datasets/Heart+Disease.
